# 10 years of 25-hydroxyvitamin-D testing by LC-MS/MS-trends in vitamin-D deficiency and sufficiency

**DOI:** 10.1016/j.bonr.2018.05.003

**Published:** 2018-05-23

**Authors:** Kornelia Galior, Hemamalini Ketha, Stefan Grebe, Ravinder J Singh

**Affiliations:** a Department of Laboratory Medicine and Pathology, Mayo Clinic, Rochester, MN, United States; b Department of Pathology, University of Michigan Health System, Ann Arbor, MI, United States

**Keywords:** Vitamin D supplementation trends, LC-MS/MS, Vitamin D toxicity

## Abstract

In early 2000's vitamin-D deficiency was shown to be prevalent in several countries including the United States (US). Studies exploring the role of vitamin-D metabolism in diverse disease pathways generated an increased demand for vitamin-D supplementation and an immense public interest in measurement of vitamin-D metabolite levels. In this report, we review the role of vitamin-D metabolism in disease processes, clinical utility of measuring vitamin-D metabolites including 25-hydroxyvitamin-D (25(OH)D), 1,25-dihydroxyvitamin-D and 24,25-dihydroxyvitamin-D and discuss vitamin-D assay methodologies including immunoassays and liquid chromatography mass spectrometry (LC-MS/MS) assays. We also provide examples of vitamin-D toxicity and insight into the trends in serum 25(OH)D levels in the US population based on 10 years of data from on serum 25(OH)D values from ~5,000,000 patients who were tested at the Mayo Medical Laboratories between February 2007–February 2017.

## 1 Case histories

### 1.1 Vitamin-D toxicity due to intentional vitamin-D overdose

In 2015, we reported a pediatric case of vitamin-D intoxication with vitamin-D_3_ supplements (Ketha et al., 2015). Briefly, four-month-old infant was admitted to Mayo Clinic for significant dehydration, lethargy, and weight loss. Routine chemistry blood panel revealed total calcium levels of 18.7 mg/dL indicating severe hypercalcemia. PTH levels were suppressed (<6 pg/mL; normal range: 15–65 pg/mL) and serum phosphorous was 1.9 mg/dL; normal range: 2.5–4.5 mg/dL. Further medical evaluation revealed hypercalciuria and nephrocalcinosis. During the discussion with the mother it was discovered, that in the last two months the infant was receiving daily dosage of oral vitamin-D_3_ supplementation that was greater than the manufacturer's recommendation. It was estimated that the baby was receiving ~50,000 IU of vitamin-D per day while recommended dose on the label was 2,000 IU. It was also determined that the actual amount of vitamin-D per drop was 6,000 IU, 3 fold higher than what it was stated on the label (2,000 IU). Vitamin-D metabolites were tested in the infant's blood and were as follows: 25(OH)D_3_, 293 ng/mL (optimal range of total 25(OH)D:20–50 ng/mL); 1,25(OH)_2_D_3,_ 138–111 pg/mL (optimal range: 24–86 pg/mL); ratio of 24,25(OH)_2_D_3_ to 25(OH)D_3_ was 0.11–0.14 (normal range: 0.07–0.18) and suggesting normal Cyp24A1 function. Baby was treated with fluids and calcitonin to reverse hypercalcemia and lower vitamin-D levels and both biomarkers reached the normal levels within 3 months.

### 1.2 Vitamin-D toxicity due to manufacturing error

In 2013, Kara et al. also described vitamin-D toxicity in children, but in her cases the error was exclusively caused by the manufacturer producing fish oil supplements with concentration of vitamin-D that was 4,000 times the concentration stated on the label (Kara et al., 2014). Seven children, below age 4.2, were admitted to the hospital due to significant hypercalcemia (median serum Ca was 16.5 mg/dL; range: 13.4–18.8; reference range: 8.8–10.8 mg/dL). Serum phosphorous and PTH levels were found to be in normal range. All children presented with similar clinical manifestations: vomiting, dehydration, constipation and weight loss. Median concentration of serum 25(OH)D was 620 ng/mL (range: 340–962 ng/mL; reference range: 30–80 ng/mL). It was later estimated that the children were receiving daily vitamin-D_3_ dosage between 266,000 IU and 8,000,000 IU, which was significantly above the normal recommendation (2,000 IU). Calcium levels in all children were corrected within 3 days (range: 2–7 days) and vitamin-D levels normalized within 3 months.

### 1.3 Vitamin-D toxicity from overcorrecting vitamin-D deficiency

In 2015, Kaur et al. described 16 patients who overdosed with vitamin-D supplements to treat their vitamin-D deficiencies (Kaur et al., 2015). All patients presented with similar symptoms of vitamin-D toxicity: vomiting, weight loss, nausea and constipation. Upon admission, patients were noted to have median (range) serum calcium level of 13.0 mg/dL (11.1–15.7 mg/dL), median (range) serum 25(OH)D of 371 ng/mL and normal phosphorous and PTH results. It was determined that patients were taking ~77,000 IU of vitamin-D_3_ each day over a period of 4–7 weeks and this mega-dose resulted in vitamin-D toxicity in all patients.

## 2 Background

Vitamin-D and parathyroid hormone (PTH) are the principle regulators of calcium homeostasis in all tetrapods and play an important role in bone metabolism (Lips and van Schoor, 2011; Pettifor and Prentice, 2011; Rizzoli, 2014). Vitamin-D exists in two major forms, vitamin-D_2_ (ergocalciferol) and vitamin-D_3_ (cholecalciferol). Both are formed by UV irradiation of either 7-dehydroergosterol (phytoplankton, fungi and yeast) or 7-dehydrocholesterol (all vertebrates with the exception of fish [predominately dietary-derived vitamin-D_2_]). The two forms differ only in the substitution on the side chain. It is believed that vitamin-D_2_ first emerged in phytoplankton about 750 million years ago and served as the vitamin-D source for marine vertebrates until the transition to terrestrial life occurred and vitamin-D_3_ production commenced in the skin of tetrapods (Holick, 2008a).

**Fig. 1:**
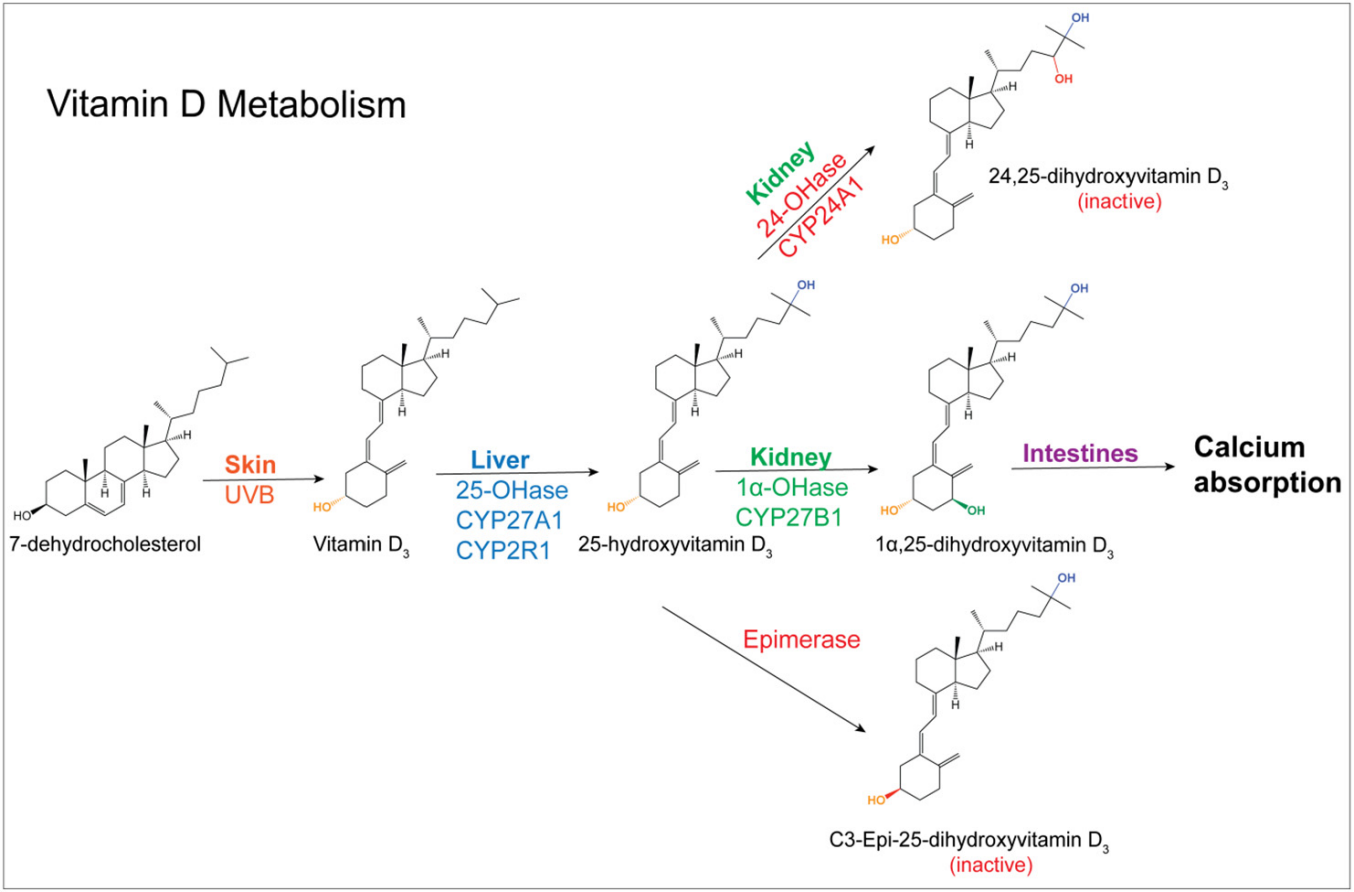
The vitamin-D metabolic pathway.

In modern humans, primary sources of vitamin-D include diet or supplements (vitamin-D_2_ and/or-D_3_) and vitamin-D_3_ derived from 7-dehydrocholesterol in the skin after exposure to UV light (Fig. 1) (Holick, 2008b; Singh, 1985). Endogenous or dietary vitamin-D binds to vitamin-D binding protein and is transported to the liver, where it is hydroxylated at the carbon 25-position, creating 25-hydroxyvitamin-D (25(OH)D). 25(OH)D is the most abundant circulating form of the vitamin-D. Vitamin-D and 25(OH)D have no established bioactivity and can be viewed as pro-hormones.

25-hydroxyvitamin-D-1α-hydroxylase (CYP27B1) in the kidneys converts 25(OH)D to the active hormone, 1,25-(OH)_2_D (Figure 1). 1,25-(OH)_2_D belongs to the superfamily of thyroid- and steroid hormones, and exerts its effects through changes in gene transcription. It binds to a nuclear receptor (Vitamin-D receptor, VDR) that dimerizes with the retinoid X receptor (RxR) before binding to gene regulatory DNA elements. There are between 2000 and 8000 VDR response elements in the human genome. Transcriptional response depends on the number of available VDR and RxR, the concentrations of their respective ligands, the nature of the response element (enhancing or inhibiting), availability of co-factors and transcriptional accessibility of the respective genes (Pike, 2016).

Consequently, the actions of 1,25-(OH)_2_D across various tissues of an entire animal are complex and in most cases require detailed study to detect clinically relevant changes. The areas that are exceptions to this rule are calcium and phosphate metabolism and bone metabolism. 1,25-(OH)_2_D obvious principle role is (i) to increase intestinal calcium absorption, (ii) to increase calcium and phosphate reabsorption in the kidneys, (iii) to increase calcium release from bones in concert with PTH, and (iv) to downregulate PTH production. The activity of the CYP27B1 in turn is regulated by PTH and other factors including calcium demand.

Due to these feedback mechanism, production of 1,25(OH)_2_D remains constant over a wide range of serum 25(OH)D concentrations, with excess 25(OH)D and 1,25(OH)_2_D being converted to inactive metabolites, 24,25(OH)_2_D and 1,24,25(OH)_3_D by 25-hydroxyvitaminD-24-hydroxylase (CYP24A1). Additional, independent inactivation is catalyzed by 3-epimerase, which isomerizes the C-3 OH group of 25(OH)D and 1,25(OH)_2_D from the α to β orientation, reducing bioactivity (Bikle, 2014).

## 3 Role of vitamin-D metabolite measurement in health and disease

Since vitamin-D_3_ and vitamin-D_2_ are either stored in adipose tissue or rapidly metabolized to the corresponding 25-hydroxylated metabolites, their serum levels fluctuate widely and there is no clinical value in monitoring these forms of vitamin-D in the circulation.

Among the >40 vitamin-D metabolites discovered so far, three have been shown to be the most clinically relevant: 25(OH)D, 1,25(OH)_2_D and 24,25(OH)_2_D.

25(OH)D serum concentrations are useful for assessing vitamin-D reserves. 25(OH)D levels increase steadily upon exposure of skin to UV-containing light or after consuming supplements containing cholecalciferol or ergocalciferol. In recent times, many investigators have explored associations between vitamin-D metabolism and cardiovascular disease, obesity, cancer, and autoimmune diseases (Delvin et al., 2014; Wang, 2009; Kulie et al., 2009; Holick, 2007); however, no clear recommendations for clinical interventions have yet emerged from these studies. By contrast, studies of bone health and 25(OH)D levels have at least in part resulted in clinically useful, albeit not always uncontested, conclusions. One study, which investigated the effect of calcium and vitamin-D (cholecalciferol) supplementation on bone density in men and women older than 65 years, found a moderate reduction in bone loss in the femoral neck, spine, and total body over the three-year study period and a reduced incidence of non-vertebral fractures in the group supplemented with 500 mg of calcium plus 700 IU of vitamin D3 (cholecalciferol) per day compared to placebo (Dawson-Hughes et al., 1997). Vitamin-D supplementation was also shown to reduce the risk of falls by >20% among ambulatory or institutionalized older individuals with stable health (Bischoff-Ferrari et al., 2004). Benefits of vitamin-D supplementation on fracture prevention are related to its effect on intestinal calcium absorption and bone mineral density (Bikle, 2012). However, in years that followed, the findings for falls and fractures in the elderly were mixed when supplementation with intermittent single large doses of vitamin-D (50,000–100,000 IU) was examined in randomized controlled trials (RCTs). A meta-analysis of nine RCTs found that supplementation with intermittent, high dose vitamin-D might not be effective in preventing overall mortality, fractures, or falls among older adults (Zheng et al., 2015). The paradoxical increase in fracture-risk in some of the reviewed studies has been hypothesized to be caused by an up-regulation of the CYP24A1 enzyme and an increased clearance of 1,25(OH)_2_D.

**Fig. 2:**
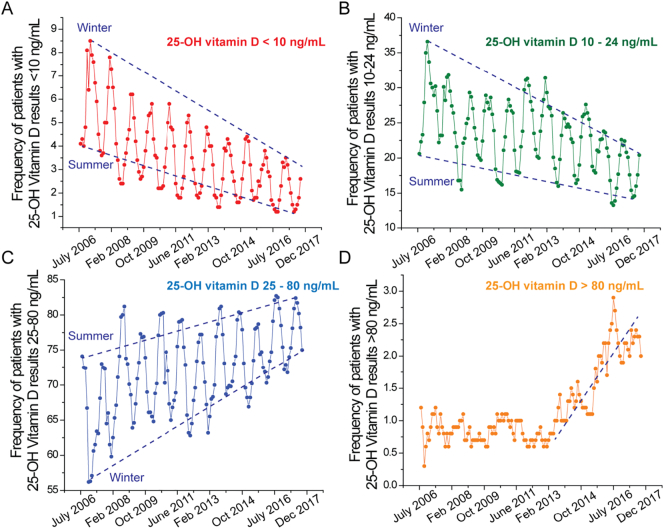
Frequency of patients with different 25-OH vitamin-D concentrations collected between July 2006 and December 2017. A) Deficiency; B) Insufficiency; C) Sufficiency; D) Toxicity.

The Institute of Medicine has recommended that at the low end a serum 25(OH)D level of at least 20 ng/mL is sufficient for 97.5% of the population for effective prevention of bone disease and fractures, while a level of 50 ng/mL is considered as a safe upper healthy population cut-off. Serum 25(OH)D levels <20 ng/mL represent deficiency (Fig. 2). For every 100 IU of vitamin-D supplement administered, the 25(OH)D levels rise by 0.5 to 1 ng/mL. Deficiency of 25(OH)D can cause bone pain and muscle weakness, and in extreme cases osteomalacia in adults and rickets in children. However, mild deficiency may not necessarily be associated with overt symptoms. On the other end of the spectrum, sustained levels of 25-OH-vitamin D > 50 ng/mL might lead to hypercalciuria, stone formation and ultimately decreased renal function. Frank vitamin-D toxicity might occur at even higher levels and is characterized biochemically by hypercalcemia, hyperphosphatermia, suppressed serum PTH concentrations, and markedly elevated 25(OH)D levels. Clinical manifestations of severe toxicity include vomiting, nausea, abdominal pain, fatigue, and weakness, and sometimes rapidly developing nephrocalcinosis (Misselwitz et al., 1990).

The tight regulation of 1,25(OH)_2_D production means that its levels fluctuate little. Except in cases of extreme vitamin-D deficiency or toxicity 1,25(OH)_2_D serum concentrations typically remain 22–65 pg/mL. Therefore, serum 1,25(OH)_2_D measurements are in the main only indicated when there is suspicion of unregulated conversion of 25(OH)D to 1,25(OH)_2_D, as might be seen in some granulomatous diseases, or if patients have advanced renal impairment with or without 1,25(OH)_2_D replacement therapy.

A combination of measurements of serum 25(OH)D, 1,25(OH)_2_D, 24,25(OH)_2_D and C3-epi-25(OH)D might be necessary in the differential diagnosis of acquired- (e.g. granulomatous diseases), iatrogenic- (e.g. over-supplementation) and genetic causes (e.g. 24-hydroxylase deficiency) of non-PTH driven hypercalcemia. In recent years, 24,25(OH)_2_D has received a lot of attention in this context. It is a marker for CYP24A1 function when used in conjunction with 25(OH)D measurements. In normal individuals, 24,25(OH)_2_D is 7–35% of the total 25(OH)D (Ornoy et al., 1978). A 25(OH)D/24,25(OH)_2_D ratio of 99 or greater is indicative of CYP24A1 deficiency.

Once the cause of vitamin-D toxicity has been established, serum vitamin-D levels might need to be monitored until they fall to about 30–50 ng/mL.

## 4 Vitamin-D assays

Methodologies for 25(OH)D measurements include high performance liquid chromatography (HPLC), radioimmunoassay (RIA), automated immunoassays and liquid chromatography-tandem mass spectrometry (LC-MS/MS), while current 1,25(OH)_2_D and 24,25(OH)_2_D measurements involve RIAs or LC-MS/MS. Analytical challenges have been reported for all of these methods (Singh, 2008), but currently measurement of vitamin-D compounds by HPLC with MS/MS detection (Heijboer et al., 2012; Eisman and Deluca, 1978) has been established as the gold standard for vitamin-D metabolite testing

Despite this, automated immunoassays are the mainstay for the majority of high volume analytes in clinical laboratories. They offer high throughput and automated sample handling and require minimal manual labor. Consequently, 90% of routine 25(OH)D analyses today are performed by automated immunoassay. However, most automated immunoassays suffer from the inherent narrow dynamic range and specificity limitations of competitive immunoassays. The former results in these assays frequently either underestimating or overestimating 25(OH)D concentrations at both the low and the high end of their measurement range, i.e. precisely at the concentrations where accuracy would be most important (Kocak et al., 2015; Holmes et al., 2013; Farrell et al., 2012), while the latter manifests itself as unequal affinity for 25(OH)D_2_ versus 25(OH)D_3_, and occasional interferences (Tolan et al., 2017). In concert, these issues also result in significant differences between the results of different immunoassays. The discordance between 25(OH)D values from different assays is magnified by differences in standardization of each assay (Snellman et al., 2010).

LC-MS/MS overcomes these issues and has become widely accepted for routine use for many low molecular weight analytes in clinical laboratories due to improved analytical specificity and sensitivity and wider dynamic range compared to immunoassay methods (Muller and Volmer, 2015; El-Khoury et al., 2011; Singh, 2010). LC-MS/MS vitamin-D assays offer better accuracy at medical decision levels to correctly classify patients as vitamin-D deficient and sufficient. Several LC-MS/MS methods for measurement of all clinically relevant metabolites of vitamin-D including 25(OH)D (Thacher et al., 2010; Singh et al., 2006), 1,25(OH)_2_D (Hoofnagle et al., 2010; Strathmann et al., 2011) and 24,25(OH)_2_D (Ketha et al., 2016) have been reported. However, although LC-MS/MS is considered the gold standard for 25(OH)D testing, according to the Accuracy-Based Vitamin-D 2016 Survey, only 74 out of 364 US laboratories used LC-MS/MS for 25(OH)D testing. Initial capital investment, the labor and time intensive nature of development and implementation of clinical LC-MS/MS assays and slower turnaround time might be the keys impediments towards a global adoption of LC-MS/MS for vitamin-D metabolite quantitation (Grebe and Singh, 2011).

The College of American Pathologists (CAP) and Vitamin-D External quality assessment scheme (DEQAS) surveys are used to monitor the performance of laboratories using various methods for testing of 25(OH)D (Burdette et al., 2017). The survey feedback does not assess the accuracy of 25(OH)D measurements by laboratories, but scores laboratories for agreement within the group using a particular method. Additionally, lack of standardization has been recognized as a challenge in steroid hormone testing. The Center for Disease Control and Prevention has established a Vitamin-D Standardization Certification Program (VDSCP) focused on providing reference measurements for 25(OH)_2_D, to assess the accuracy and precision of vitamin-D tests, to monitor their performance over time, and provide technical support to external quality assurance programs, proficiency testing programs, and research studies (VDSCP: Vitamin-D Standardized-Certification Program). A recent study by VDSCP study established core performance criteria, namely CV ≤ 10% and mean bias ≤5% for 25(OH)D quantitation. Inter-laboratory performance of 25(OH)D measurement was compared by providing a set of 50 individual donor samples to 15 laboratories representing national nutrition survey laboratories, assay manufacturers, and clinical or research laboratories. Samples were analyzed using immunoassays and LC-MS/MS. All LC-MS/MS results achieved VDSCP criteria, whereas only 50% of immunoassays met the criterion for a ≤ 10% CV and only three of eight immunoassays achieved the ≤5% bias (Wise et al., 2017). Perhaps some of these issues might be addressed with the availability of National Institute of Standards and Technology (NIST) standard reference materials (Burdette et al., 2017; Thienpont et al., 2012), but even then the inherent problems of immunoassays will likely continue to negatively impact vitamin-D laboratory testing quality.

## 5 Decreasing vitamin-D deficiency in the US – lessons learnt from 10 years of LC-MS/MS 25(OH)D testing

During the first decade of the 21st century, Vitamin-D deficiency was shown to be highly prevalent in the USA (Holick, 2007). In the years that followed, several studies echoing these findings were reported. Consequently, the current decade has witnessed an increased public awareness of vitamin-D supplementation.

The clinical laboratory at the Mayo Clinic in Rochester, MN is a referral laboratory for a vast network of clinical providers in the US. During the 2007–2017, ~5,000,000 patient samples were tested for 25(OH)D by LC-MS/MS in our laboratory (Netzel et al., 2011). Patient results were categorized into the following groups <10 ng/mL, 10–24 ng/mL, 25–80 ng/mL and > 80 ng/mL. Frequencies were calculated by using the formula: ((Number of patients in a 25(OH)D category / total number of patients tested in the corresponding week) ∗ 100), in order to gauge whether there have been any changes in the frequency of 25(OH)D in the four categories over time. The frequency of patients in each serum 25(OH)D category is shown in Fig. 2. Seasonal variation, as a reflection of the amount of sunlight to which a person is exposed, was observed in the <10 ng/mL, 10–24 ng/mL, 25–80 ng/mL categories. As expected, serum concentrations of 25(OH)D were highest in late summer and lowest in spring. At the end of summer of 2006 (shown in red, Fig. 2a), 4.3% of the population being tested had serum 25(OH)D levels <10 ng/mL. This number increased to 8.5% by the end of winter of 2007. After 10 years, a significantly lower percentage of the population had serum 25(OH)D levels <10 ng/mL (0.2% and 3.1% post-summer and post-winter of 2017, respectively). Similarly, the percentage of patients with 25(OH)D range of 10–24 ng/mL decreased steadily between 2007 and 2017 (green, Fig. 2b). By contrast, the percentage of patients with 25(OH)D levels (between 25 and 80 ng/mL) increased from 72.5% to 82.4% post-summer and from 60.6 to 72.9% post-winter during the ten-year period (blue, Fig. 2c).

Of note, the percentage of patients with levels >80 ng/mL remained constant (1.1%), without seasonal variation until the end of 2012 (Fig. 2d). Since then, it has been slowly increasing to 2–2.5% probably due to an increased awareness of vitamin-D deficiency and an increased use of over-the-counter supplements and prescriptions of high-dose vitamin-D. Given that our 25(OH)D vitamin-D assay is a laboratory developed test, we have verified that there were neither a shifts in the calibration over time, nor biases and trends in quality controls nor biases due to reagent lot-to-lot changes. Based on our data obtained with this assay of demonstrably stable analytical performance, it appears that vitamin-D supplementation is driving improvements in population 25(OH)D levels. Our findings are consistent with a recent study which also found modest increases in the serum 25(OH)D concentrations in the US population from 1988 to 2010 (Grebe and Singh, 2011).

The observation that serum 25(OH)D levels in the general US population have increased during the last decade has important implications. Population based and basic science studies exploring the role of vitamin-D metabolism in health and disease pathways have raised public awareness about effective modes of vitamin-D supplementation. Of note, the increase in the percentage of patients who have circulating concentration of 25(OH)D of >80 ng/mL warrants further attention. Long-term physiological effects of persistently elevated 25(OH)D may need to be investigated in clinical studies.

## 6 Conclusion

Over the last decade, the frequency of measurements of 25(OH)D in the healthy population has significantly increased due to an increased awareness of vitamin-D deficiency and its potential association with many diseases beyond its role in maintaining bone health. This also resulted in an increased demand for vitamin-D metabolite measurements. Quantification of 25(OH)D in serum is the best indicator of vitamin-D status of individuals. 25(OH)D accurately reflects the body's vitamin-D stores. At present, LC-MS/MS assays offer the best accuracy for vitamin-D metabolite analysis. Accurately measuring vitamin-D metabolite levels is important to classify patients as vitamin-D deficient so appropriate supplementation can be recommended. Monitoring patients who receive high-dose vitamin-D supplementation is also of clinical value. Our retrospective study has shown seasonal changes of 25(OH)D as well as an overall change in frequency of patients with vitamin-D deficiency and sufficiency. Of note our data show that the frequency of patients with 25(OH)D deficiency decreased over the last decade (2007–2017). The flip side of this is that the percentage of the patients undergoing 25(OH)D testing who have serum 25(OH)D > 80 ng/mL has slowly increased during that time. While the proportion of patients with potentially toxic levels remains relatively low, the consequences of toxicity can be severe, and prospective population studies to investigate clinical impact and long-term safety of higher circulating levels of 25(OH)D might be warranted.

## Conflict of interest

I have no conflict of Interest for the data in this publication.
